# Microbial communities inhabiting shallow hydrothermal vents as sentinels of acidification processes

**DOI:** 10.3389/fmicb.2023.1233893

**Published:** 2023-09-01

**Authors:** Erika Arcadi, Carmen Rizzo, Rosario Calogero, Valentina Sciutteri, Francesco Fabiano, Pierpaolo Consoli, Franco Andaloro, Teresa Romeo

**Affiliations:** ^1^Department of Biology and Evolution of Marine Organism, Stazione Zoologica Anton Dohrn, Sicily Marine Centre, Messina, Italy; ^2^Marine Biotechnology Department, Stazione Zoologica Anton Dohrn–, Sicily Marine Centre, Messina, Italy; ^3^Institute of Polar Sciences, National Research Council (CNR-ISP), Messina, Italy; ^4^Department of Integrative Marine Ecology, Stazione Zoologica Anton Dohrn, Sicily Marine Centre, Messina, Italy; ^5^Department of Biology and Evolution of Marine Organisms, Stazione Zoologica Anton Dohrn, Sicily Marine Centre, Messina, Italy; ^6^National Institute for Environmental Protection and Research, Milazzo, Italy

**Keywords:** shallow hydrothermal vents, acidification effects, microbial communities, redox potential, extremophiles

## Abstract

**Introduction:**

Shallow hydrothermal vents are considered natural laboratories to study the effects of acidification on biota, due to the consistent CO_2_ emissions with a consequent decrease in the local pH.

**Methods:**

Here the microbial communities of water and sediment samples from Levante Bay (Vulcano Island) with different pH and redox conditions were explored by Next Generation Sequencing techniques. The taxonomic structure was elucidated and compared with previous studies from the same area in the last decades.

**Results and discussion:**

The results revealed substantial shifts in the taxonomic structure of both bacterial and archaeal communities, with special relevance in the sediment samples, where the effects of external parameters probably act for a long time. The study demonstrates that microbial communities could be used as indicators of acidification processes, by shaping the entire biogeochemical balance of the ecosystem in response to stress factors. The study contributes to understanding how much these communities can tell us about future changes in marine ecosystems.

## Introduction

Substantial changes in terms of warming and acidification are affecting the marine world, with effects that we are not yet able to fully predict. A changing climate means a changing ocean with increasing temperatures, rising the sea levels and changing ocean chemistry (e.g., ocean acidification), with far-reaching future impacts (Reinold et al., 2019). Ocean warming and acidification act simultaneously and jointly, as the ocean warming determines the increase of stratification in the water column that prevents the nutrient transport to the upper layers, while pH decrease alters the marine carbonate equilibrium by increasing the CO_2_ concentration and a decreasing the carbonate concentration. Consequently, climate change and ocean acidification strongly affect the growth and survival of marine organisms (Brander, 2010; Joint et al., 2011; Hollowed et al., 2013), acting as main driving forces for local adaptation/acclimatization, physiological plasticity and evolutionary changes. The challenge for many researchers is to elucidate how marine species can respond via acclimatization or adaptation to acidification, and how fast their response occurs.

Only recently the importance of using environmental acidified systems, such as CO2 vents, as natural laboratories for addressing effects of ocean chemistry changes on marine ecosystems has been recognized (Boatta et al., 2013; González-Delgado and Hernández, 2018; Aiuppa et al., 2021). Due to their limits in extension and depth and their variability in space and time regarding pH/carbonate chemistry, the natural CO2 vents cannot be considered precise analogues to study the ocean acidification problem at a large and global scale, however they can provide useful information on the effects of long-term exposure to high pCO2 and low pH on several organisms. Until now most studies were addressed to the impact of climate change on eukaryotes (Apostolaki et al., 2014; Rizzo et al., 2022 and therein references), while the contribution and susceptibility of microorganisms to a changing climate was less investigated. This lack of information is a very serious gap since microbial communities have a recognized pivotal role in the balances and dynamics of marine ecosystems, as they play a key role in reshaping the oceans and atmosphere and supporting all higher trophic level organisms (Cavicchioli et al., 2019). The microbial communities inhabiting shallow hydrothermal vents are involved in organic matter synthesis, breakdown or mineralization, metal cycles and interactions with fauna (Bellec et al., 2020). Indeed, primary production at shallow-water hydrothermal vents is based mainly on both photosynthesis and chemosynthesis processes, with variable contributions depending on the characteristics of vent emission. Chemoautotrophs contribute to the transfer of energy from the geothermal source to the higher trophic levels by using several chemosynthetic pathways such as sulfur-oxidation, nitrification, etc. (Sievert and Vetriani, 2012). The microbial communities inhabiting deep and, to a lesser extent, also shallow hydrothermal vents, are affected by the strongly negative redox potential and low pH. According to Joint et al. (2011), the natural pH variations in aquatic environments are well tolerated by microorganisms with respect to negative redox potential and might not dramatically affect microbial communities. As known, marine microbes are able to respond rapidly to environmental shifts (Hagström et al., 2002) and they play a key role in oceanic biogeochemical cycles (Fuhrman et al., 2015).

Mediterranean hydrothermal vent systems are principally localized in the Aegean Arc and Aeolian Arc (Rizzo et al., 2022). These peculiar ecosystems include many diversified habitats, mainly on arc volcanoes, back-arc basins, and hot spot volcanoes. They are characterized by a range of 0–1,200 m depth, with temperatures comprised between 13 and 135°C. Some sites can receive a larger amount of terrigenous sediments as they are located more closely to the land, but they all share the presence of well-note gaseous emissions (CO_2_, H_2_S, CH_4_). Not all Mediterranean vents are characterized by hot emissions, as revealed by the low temperature value (between 13 and 25°C) detected in the deep Ischia Island, Palinuro Cape, in some areas of the Panarea Volcanic Complex, Yali, Methana, Nisiros, Santorini and Kos hydrothermal vents (Rizzo et al., 2022).

Among the hydrothermal sites of the Aeolian Arc, Vulcano Island has been identified as a natural laboratory to study the effects of acidification on biodiversity, with the advantage of shallow depths in Levante Bay which make the sites easy to reach for constant investigations. In the southern part of the bay, bubble gasses are mainly characterized by a variable concentration of H_2_S (1.57–2.47 vol%, Carapezza et al., 2011) probably derived by alkaline hydrolysis of metal sulfides promoted by weakly acidic waters (Capaccioni et al., 2001). Previous surveys conducted in the area showed that H_2_S concentrations decrease moving away from the emission points, in particular from the main vent and only a small portion of the gas enters into the aqueous phase where it oxidizes to sulfate due to the high O_2_ saturation recorded in the bay, especially in the northern area (Boatta et al., 2013).

Over the years various studies have been conducted to define the taxonomic structure of the microbial communities in relation to the natural acidification in marine hydrothermal vents in the Aeolian Arc (Gugliandolo and Maugeri, 1993, 1998; Maugeri et al., 2002; Gugliandolo et al., 2003), with highly diversified observations. According to Kerfahi et al. (2014), the taxonomic structure of bacterial communities inhabiting sediments showed variations in the abundance of few taxa in relation to long-term acidification at Vulcano vents (i.e., viz. *Georgenia*, *Lutibacter*, *Photobacterium*, *Acinetobacter*, and *Paenibacillus* members), but overall the main taxa maintained their abundance ratio also under the acidified conditions. Based on these observations, and on the awareness that pH variations naturally occur in the aquatic environments and are well tolerated by microorganisms, some authors argue that overall the degree of pH variations due to anthropogenic ocean acidification might not dramatically affect microbial communities (Joint et al., 2011; Roy et al., 2013; Oliver et al., 2014). In contrast, Oliver et al. (2014) make a clear distinction between oxic acidified sediments and acidified sediments with negative redox potential. They have shown that under acidified conditions there are no variations in abundance, structure and composition of the microbial community, while bacterial communities associated with oxic sediments appear to significantly differ at sites with naturally low pH compared with control sites (Raulf et al., 2015; Hassenrück et al., 2016).

Several and relatively recent studies were conducted on the composition of the microbial communities of hydrothermal vents of Vulcano (Gugliandolo and Maugeri, 1998; Amend et al., 2003; Rusch et al., 2005; Fagorzi et al., 2019; Aiuppa et al., 2021) and also assessed their possible correlation with the composition of the gas emissions (Fagorzi et al., 2019). More recently, a colonization experiment was conducted in the shallow-water vent system of Levante Bay to investigate the response of microbial biofilms to ocean acidification (Sciutteri et al., 2022).

In this work, the natural pCO_2_/pH gradient in Levante Bay at Vulcano vents was used as a factor to investigate more deeply the effects of ocean acidification on the microbial communities associated with sediment and water samples. In addition, some mucilaginous and floating thickenings observed in the area, hereafter referred as “floccules,” were also collected to ascertain their microbial biofilm-like nature. The study was aimed at comparing the microbial communities of water and sediments, and exploring analogies/differences between them and the previous description, to establish if we are already assisting to a reverse of the biogeochemical status, or if prokaryotic communities are able to manage shifts in their structure in relation to the external conditions.

## Materials and methods

### Study area

Vulcano Island is the southernmost island of the Aeolian Arc, located in the Aeolian Archipelago in the Southern Tyrrhenian Sea. On the eastern coast of the island, emissions rich in CO_2_ and with variable temperatures (from ambient to about 100°C) were detected around Levante Bay. Hydrothermal fluids are characterized by considerable amounts of different gases, i.e., CO_2_, SO_2_, H_2_S, HF, and CO (La Fossa Crater, Chiodini et al., 2006) and CO_2_, CH_4_, and H_2_S (Levante Bay, Amend et al., 2003).

### Sample collection

**Figure 1:**
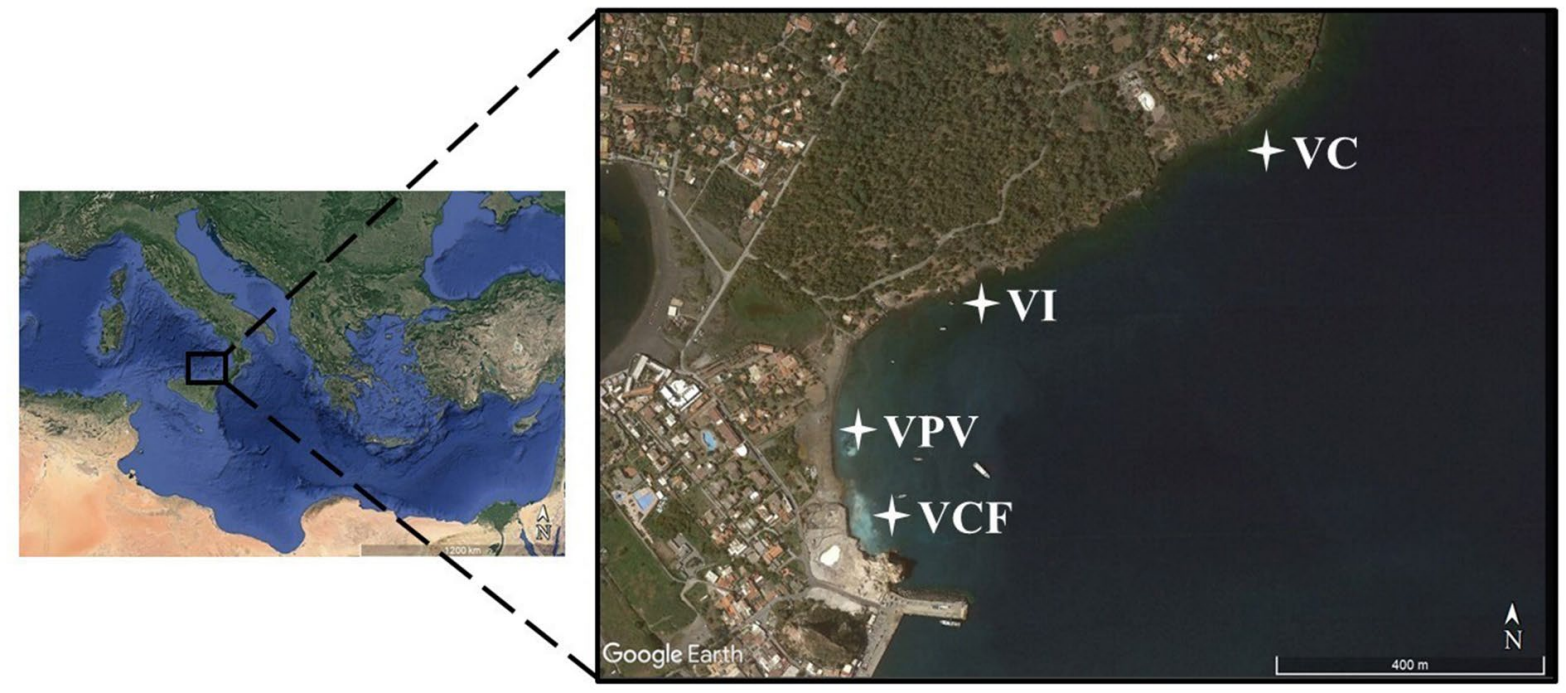
Map showing sampling area and stations (VC, Vulcano Control; VI, Vulcano Intermediate; VCF, Vulcano Campo Frizzante; VPV, Vulcano Principal Vent).

Sampling activities have been carried out at Levante Bay in Vulcano Island in June 2021 by collecting bottom water, sediment and floccule samples. Physico-chemical parameters have been measured by Multiparametric Waterproof Meter (Hanna Instruments, Romania). The four sampling sites were categorized as Vulcano Principal Vent (VPV), Vulcano Campo Frizzante (VCF), Vulcano Intermediate site (VI) and Vulcano Control site (VC, off the bay), and are shown in Figure 1. Water and sediment samples were collected using 4 L Niskin Bottles and 500 mL polycarbonate sterile bottles, respectively. Samples were stored at 4°C until processing in the laboratory. For each sampling site three samples of water and sediments have been collected. Water samples aliquots (3 L) were filtered on nitrocellulose filter (0.22 μm pore size, Millipore) and then stored at −20°C until processing. Sediment samples were directly stored at −20°C.

Floccules samples were collected in the site of Vulcano Campo Frizzante (VCF) using 50 mL syringes and then concentrated on nitrocellulose filter (0.22 μm pore size, Millipore) in duplicates and stored at −20°C until processing.

### Prokaryotic community composition

#### DNA extraction, amplification of 16S RDNA, and sequencing

The GNOME DNA kit (MP Biomedicals, United States) and the FastDNA SPIN Kit for Soil (MP Biomedicals, CA, USA) were used to extract DNA from water filters, sediments and floccules, respectively, by following the manufacturer’s instructions. The quality and concentration of all DNA extracts obtained from filters and sediments was determined by spectrophotometric measurements with a BioSpec-Nano spectrophotometer (Shimadzu Corporation). DNA samples were stored at −20°C until further analyses.

Bacterial 16S rDNA region V3-V4 was amplified for a control amplification step by using the universal primers 27F (5^′^-AGAGTTTGATCCTGGCTCAG- 3) and 907R (5′ - CCG TCA ATT CCT TTR AGT TT – 3′) on a BioRad C100 touch thermocycler (BioRad, Laboratories) in a final volume of 50 μL containing: 1 μL extracted DNA, 10 μL PCR buffer, 20 μM of each of the two primers (27F – 907R), and 0.6 μL Taq Polimerase (5 U/μL Bioline). The following program was used: an initial denaturation of 15 min at 95°C, followed by 35 cycles of 30 s 95°C, 30 s annealing at 51°C, and 30 s extension at 72°C, and a final extension 5 min at 72°C. All amplification products were verified on a 0.8% agarose-TAE gel. For sequencing, extracted DNA was used to amplify the16S rDNA region V3-V4 with the two locus specific primers with Illumina overhangs 16S-341 (5′- TCGTCGGCAGCGTCAGATGTGTATAAGAGACAGCCTACGGGNBGCASCAG −3′) and 16S-805R (5′- GTCTCGTGGGCTCGGAGATGTGTATAAGAGACAGGACTACNVGGGTATCTAATCC -3′), following the standard Illumina protocol (Kozich et al., 2013). Thirty cycles were used in the first PCR amplification with locus-specific PCR primers, while subsequent amplification that integrates relevant flow-cell binding domains and unique indices (NexteraXT Index Kit, FC-131-1001/FC-131-1002) was performed according to the protocol. Sequencing was performed using the Illumina MiSeq platform in 2×300 bp paired-end mode, following the standard protocol of the company IGA Technology Services Srl (Udine, Italy).

### Bioinformatic analysis and data elaboration

For bioinformatic analysis, FastQC was used to check the quality of raw sequences. The processing included quality filtering, trimming, de-noising and merging by using R package DADA2 to infer amplicon sequence variants (ASVs), i.e., biologically relevant variants. During the analysis, filters for reducing replicate, length, and chimera errors were also applied. Bacterial taxonomy annotation was performed using Silva database formatted for DADA2, offering an updated framework for annotating microbial taxonomy (silva_nr99_v138.1_wSpecies_train_set.fa.gz and silva_species_assignment_v138.1.fa.gz). A taxa filter was used for the decontamination of eukaryotic, chloroplast, and mitochondrial sequences. The analysis was carried out with the support of IGA Technology Services Srl (Udine, Italy). Finally, a manual inspection was done, and sequences with 0.1% abundance were not considered.

#### Scanning electron microscopy analysis

An image observation by using Scanning Electron Microscopy (SEM – FEI inspect s50) and analysis of elemental composition using the Energy Dispersive X-ray Spectrometer STR27- Bruker quantax flash 610 (software bruker esprit 2.0) were performed on floccule samples.

The Microanalysis mapping provides structural, compositional, and chemical information about sample at microscope scale. The characteristic X-rays of the elements in the sample can be recorded and discriminated on the basis of wavelength (WDS) or energy (EDS). The intensity of these characteristic radiations is proportional to the concentration of the element in the sample. Thus, X-ray microanalysis gives specific information about the composition of the elements in the sample, in terms of quantity and distribution. Briefly, the floccule sample was observed by using a scanning electron microscope with emission current 100 μA, electron high tension (EHT) at 30 kV and a working distance of 10 mm (the analysis were carried out with the support of Ambiente Lab srl, Messina).

### Statistical analysis

PERMANOVA analysis was carried out to verify the occurrence of significant differences between water and sediment communities by setting as factor the sampling site. Relative abundances obtained from taxonomic analysis were transformed and then processed to calculate Bray–Curtis similarity and perform the cluster analysis. The obtained matrix was used to perform a Non-Metric Multidimensional Analysis (nMDS). The principal component analysis (PCA) was computed by using environmental data after transformation, and by superimposing the sample site as a factor. All the analyses were performed by using the software Primer 7 (Plymouth Marine Laboratory, Rodborough, United Kingdom).

## Results

Physico-chemical parameters measured for water samples are reported in Table 1. The pH showed a gradual acidification profile from the VC toward VI, VCF and VPV, reaching a value of 6.08 at the lowest point and gradual decrease of oxygen reduction potential (ORP). The temperature ranged between 23 and 24°C, salinity was ~35 psu in all samples, the dissolved oxygen was in the range 91.3% (VPV) and 98.7% (VC).

**Table 1:** Sampling sites, coordinates, depth, and physico-chemical parameters (temperature, pH, oxide-reduction potential, electric conducibility, total dissolved solid, salinity and organic dissolved) measured at Levante Bay (Vulcano Island).

Site*	Lat.	Long.	Depth (m)	Temp. (°C)	pH	mV_pH	ORP mV	EC (μS/cm)	EC_abs (μS)	TDS (ppm)	Sal (psu)	DO (%)	DO (ppm)
VPV	38.4176	14.9599	1	24.3	6.08	n.d.	−178.9	54,050	53,300	27.02	35.75	91.3	n.d.
VCF	38.4196	14.9606	2	23.75	6.54	n.d.	−167.9	n.d.	n.d.	27	35.72	93	n.d.
VI	38.4195	14.9621	2.1	23.62	7.21	−13.8	−128.5	53,870	52,460	26.93	35.64	98.7	7.11
VC	38.4248	14.9686	5.2	23.35	8.16	66	155.5	54,080	55,470	27.04	35.72	30.0	1.99

VPV, Vulcano Principal Vent; VCF, Vulcano Campo Frizzante; VI, Vulcano Intermediate site; VC, Vulcano Control site (off the bay).

### Prokaryotic communities structure

Total sequence reads, expressed as Amplicon Sequence Variants (ASVs) and diversity indices for each site both in water and sediment are reported in Supplementary Table S1. As shown by the Shannon and Simpson index values, diversity level in microbial communities overall decreased from the control site VC to the VCF and VPV sites, with a more marked reduction in sediment with respect to water communities.

The absolute and relative abundances of Bacteria and Archaea domains referred to a total number of obtained sequences, ranging between 1,193 and 223,846. We found a total of ASVs ranging from 9,873 to 224,255 in water samples and from 4,583 to 183,326 in sediment samples. The dataset was represented by 72 phyla, 167 classes, 388 orders, 519 families, and 1,234 genera.

The average values of relative abundances for Bacteria members were 99.7 and 99.4% of the total communities for the water and sediment samples, respectively. The relative abundance of Archaea was similar in the water and sediment samples for the four sites, and increased from the control site to the VPV site especially in the sediments, even if with low percentages (1% of the total prokaryotic community).

### Bacterial microbial communities

#### Water

**Figure 2:**
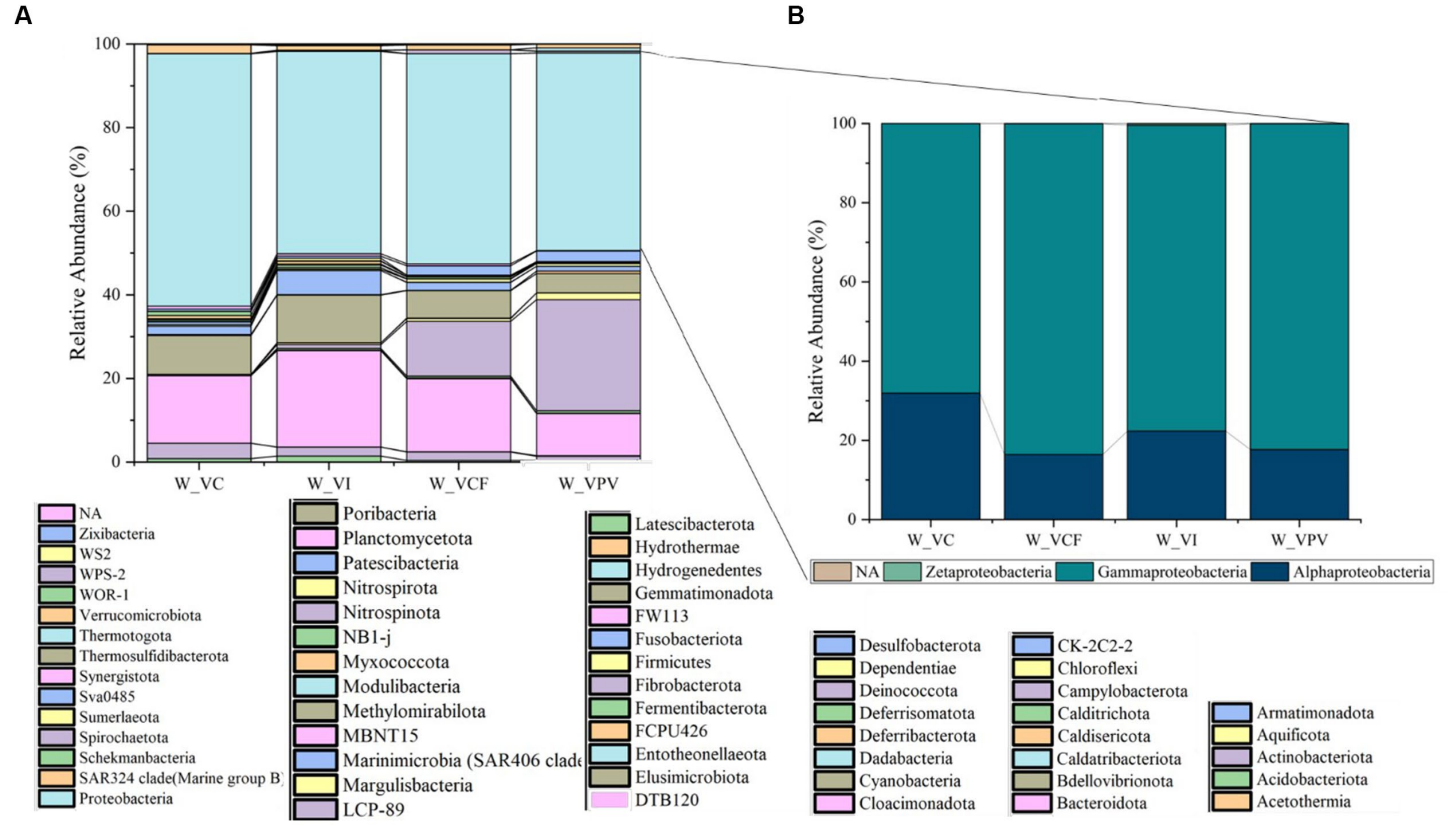
Relative abundance at phylum level **(A)** of water bacterial community from Levante Bay (Vulcano Island), with detail of Proteobacteria classes **(B)**. W_VC, Water Vulcano Control; W_VI, Water Vulcano Intermediate; W_VCF, Water Vulcano Campo Frizzante; W_VPV, Water Vulcano Principal Vent.

**Figure 3:**
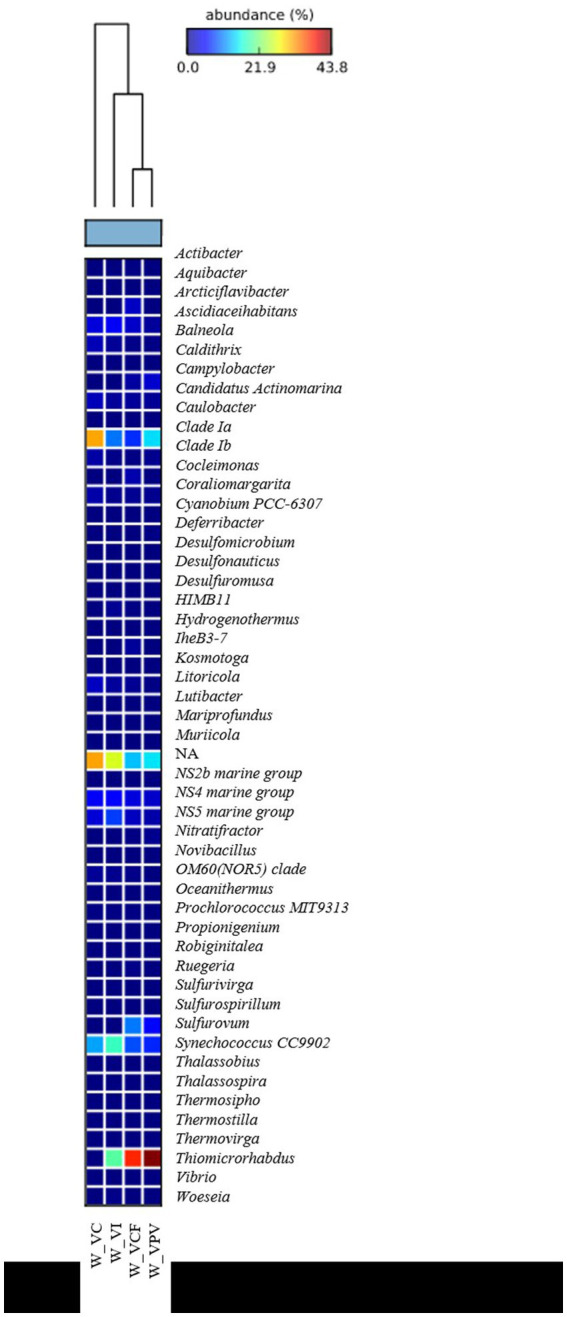
Heatmap showing taxonomic structure of water microbial communities at genus level.

As shown in Figure 2, a total of 51 and 59 different phyla were identified, respectively, in water and sediment samples using ASVs annotation. The bacterial community was mainly represented by Proteobacteria members, with average percentages ranging from 60.3% (VC) to 47.3% (VPV) of the total bacterial community. The second most represented phylum was Bacteroidota (relative average abundance ranging from 10.1% at VPV to 23% at VI of the total bacterial community), followed by Cyanobacteria (relative average abundance ranging between 4.6% at VPV and 11.4% at VI of the total bacterial community). The Phylum Campylobacterota proved to be well represented with a percentage of 26.6% (VPV) and 13.1% (VCF) of the total bacterial community, while it was poorly represented within the VI site (relative abundance <0.1%) and almost absent in VC (relative abundance <0.01%). Minor groups (average percentages ranging between 1 and 6%) were represented mainly by Desulfobacterota (1.1–5.7%), Actinobacteriota (1–3.7%), and Verrucomicrobiota (1–2%). The rest of 43 bacterial phyla was represented by groups with relative average abundance <0.1%. Among Proteobacteria classes, Gammaproteobacteria predominated in all water samples with relative average abundances ranging from 68.1% (VC) to 83.5% (VCF), followed by Alphaproteobacteria in the range between 16.4% (VCF) to 31.9% (VC), while Zetaproteobacteria and NA represented <0.1 of the total Proteobacteria (Figure 2). Bacterial sequences at the genus level in the water communities were largely represented by *Thiomicrorhabdus* affiliated to Piscirickettsiaceae family in VPV, VCF and VI sites, with a percentage of about 50% of total bacterial community, followed by *Synecococcus CC9902* in all sites with relative abundance of about 10%. *Clade Ia* is well represented in all sampled water sites, with percentages ranging from 4 to 18%, but is the most representative genus in the water samples of the control site (18%). The genus *Sulfurovum* was present in the bacterial community for the 10% of the total genera in VCF water, and 5% in VPV water. *NS4 group* was abundant with 4–5% in VC, VI and VCF water samples, and with 1% in VPV water sample, while *NS5 group* were found abundant in the VI water samples with 10%. All retrieved groups detected at the genus level are shown in Figure 3.

#### Sediment

At phylum level a total of 59 total phyla were detected in sediment samples. The most represented group was Campylobacterota, with a percentage of 39% for VCF sediments, and 57.6% for VPV sediments, while in VI and VC sediments it accounted for values of relative average abundance <1% of the total bacterial community. Overall, Proteobacteria members increased in number moving away from emission points and were represented in all sites with percentages between 14.2% (VPV) and 34.3% (VC), followed by Bacteroidota phylum with relative average abundance in the range from 6.7% (VPV) to 23% (VCF) of the total bacterial community. The abundance of Desulfobacterota ranged between 2.5% (VPV) and 18.8% (VI), while Actinobacteriota represented an average abundance range of 1.5% (VPV) – 10.1% (VC).

The phyla Calditrichota, Chloroflexi, Cyanobacteria, Firmicutes, Latescribacterota, Myxococcote, NB1, Planctomycetate, Spirochaetate, and Verrucomicrobiota groups represented, respectively, the 1–2% of total bacterial communities in all sampling sites. The remaining 44 phyla were present in the sediments with average percentages between 0.1 and 1%.

**Figure 4:**
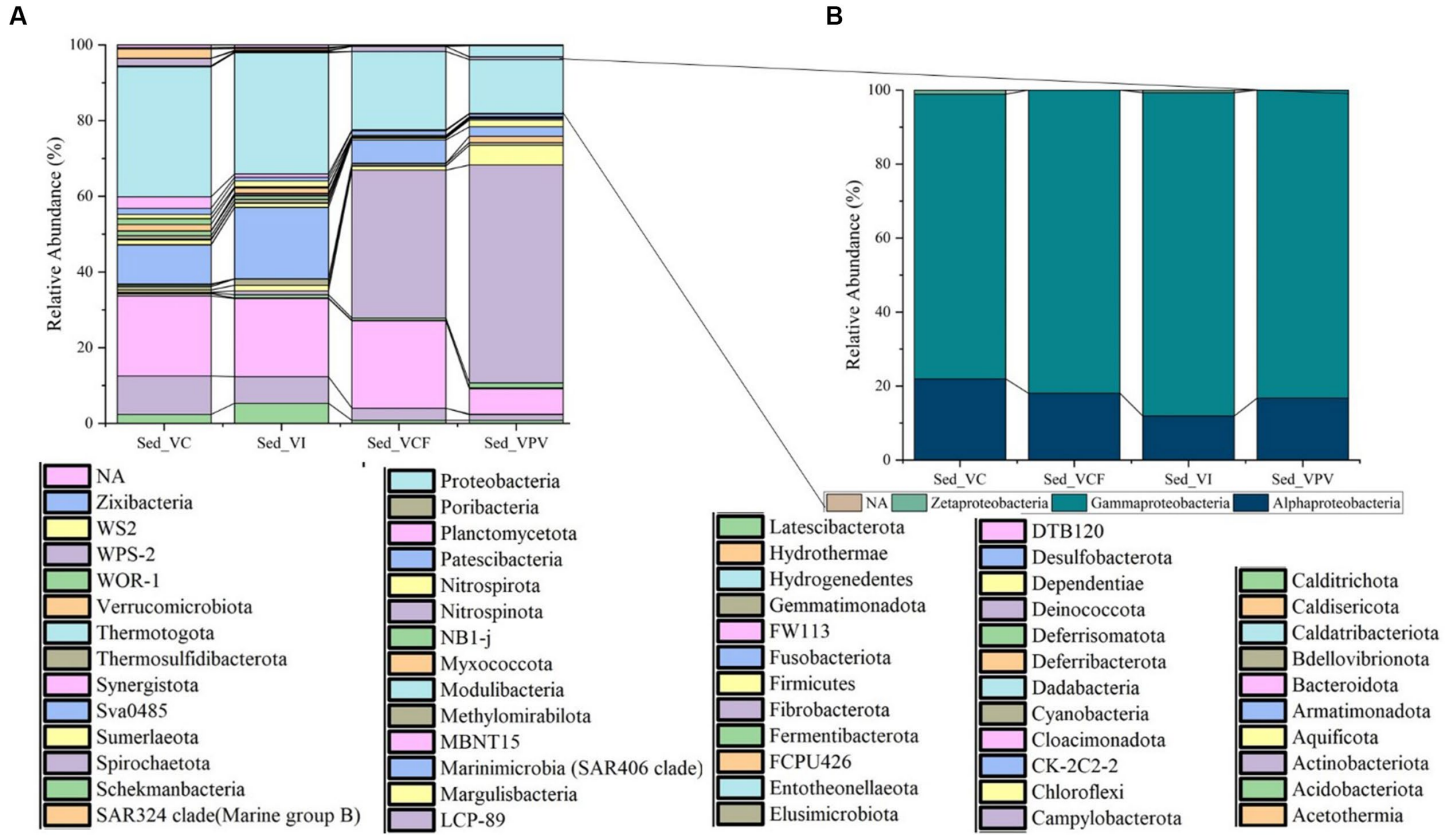
Relative abundance at phylum level **(A)** of sediment bacterial community from Levante Bay (Vulcano Island), with detail of Proteobacteria classes **(B)**. Sed_VC, Sediment Vulcano Control; Sed_VI, Sediment Vulcano Intermediate; Sed_VCF, Sediment Vulcano Campo Frizzante; Sed_VPV, Sediment Vulcano Principal Vent.

Among Proteobacteria classes (Figure 4), Gammaproteobacteria predominated achieving relative average abundances of 76.9% in VC samples, and abundance higher than 80% in all other samples. The class of Alphaproteobacteria was the second in abundance accounting for a minimum of 11.9% in VI samples up to a maximum of 21.9% in VC samples. Finally, the Zetaproteobacteria group was represented with values <0.1 of the total Proteobacteria.

**Figure 5:**
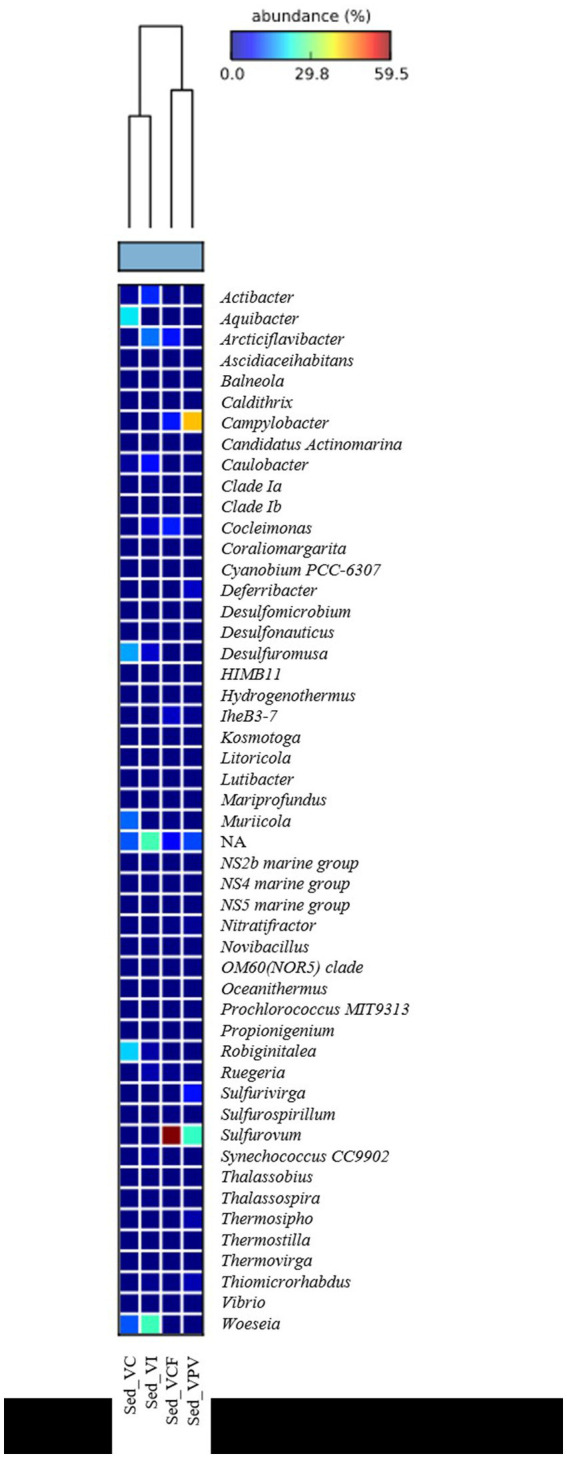
Heatmap showing the taxonomic structure of sediment microbial communities at genus level.

Bacterial sequences at genus level in the sediment (Figure 5) were mostly represented by *Campylobacter* in VPV sediment, with an average percentage of 27% of the total bacterial community, while they resulted absent in VC and VI sediment. As for the water samples, *Sulfurovum* was well represented in the VPV and VCF sediment samples with a relative average abundance of 33.6 and 22.7%, respectively. Conversely, the genus *Woeseia* was predominant in VC and VI sites, with a relative average abundance of 9.6 and 6%, respectively, while it was almost absent in VCF and VPV sites.

#### Floccules

A total of 22 classes and 119 genus were detected in floccule samples. The bacterial assemblages associated with floccules were most represented by the phylum Campylobacterota, with an average abundance of 75.5% of the total bacterial community, followed by Bacteroidota (10.4%) and Proteobacteria (8.6%). Both Desulfobacterota and Patescibacteria were found with an abundance of 1.6%, while the remaining 16 phyla were found with an abundance between 0.5 and 0.01%. More specifically 119 different genera were detected, mostly represented by *Sulfurovum* (41.8%), followed by *Nitratifractor* (23.6%), *Sulfurimonas* (7%), and *Campylobacter* (2.5%), all affiliated to Campylobacterota class. The remaining genus were instead found with an abundance <1%. Among Proteobacteria classes, Alphaproteobacteria and Gammaproteobacteria were found with an abundance, respectively, of 72.3 and 27.7% of the total Proteobacteria.

### Archaeal microbial community

#### Water

**Figure 6:**
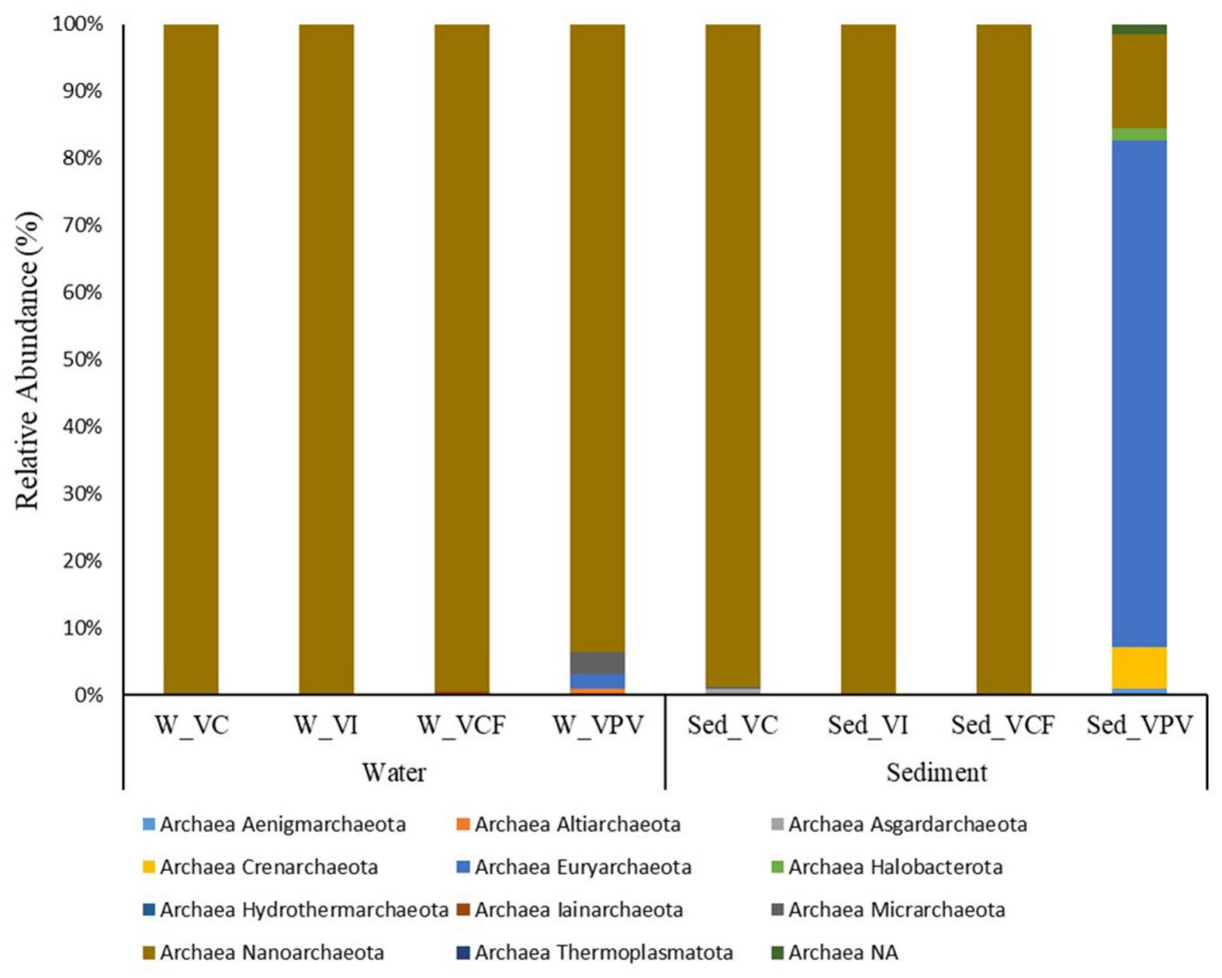
Relative abundance at phylum level of the archaeal community in water and sediment samples from Levante Bay (Vulcano Island). W_VC, Vulcano Control; W_VI, Water Vulcano Intermediate; W_VCF, Water Vulcano Campo Frizzante; W_VPV, Water Vulcano Principal Vent; Sed_VC, Sediment Vulcano Control; Sed_VI, Sediment Vulcano Intermediate; Sed_VCF, Sediment Vulcano Campo Frizzante; Sed_VPV, Sediment Vulcano Principal Vent.

Archaeal microbial community in the water samples was represented by five different phyla, namely Altiarchaeota, Euryarchaeota, Iainarchaeota, Micrarchaeota, and Nanoarchaeota (Figure 6). Nanoarchaeota was the most abundant phylum, showing percentages of relative abundance between 93.5 and 100% of the totality of the archaeal community. The archaeal communities in VC and VI sites were totally represented by Nanoarchaeota members, while the VCF and VPV sites were more diversified, with the presence of Nanoarchaeota (99.4%) and Micrarchaeota (3.3%) in VCF water, and Nanoarchaota (93.5%), Euryarchaeota (2.1%), and Altiarchaeota (1.0%), in VPV water.

No sequences were assigned at genus level in the water samples from VC, VI and VFC, while for the VPV water samples the genera *Thermococcus*, *AR15,* and *Candidatus Altiarchaeum* accounted for 1%.

#### Sediment

Sediment archaeal microbial communities were represented by 11 different phyla, respectively Aenigmarchaeota, Altiarchaeota, Asgardaarchaeota, Crenarchaeota, Euryarchaeota, Halobacterota, Hydrothermarchaeota, Iainarchaeota, Micrarchaeota, Nanoarchaeota, and Thermoplasmatota (Figure 6). While the totality of the Archaea population in the VCF and VI sites is dominated by the Phylum Nanoarchaeota, the VC site showed the concomitant presence of Nanoarchaeota (98.8% of the total archaeal community) and Asgardaarchaeota (1% of the total archaeal community). VPV was the most diversified site, with 8 different phyla, listed below in order of relative average abundance: Euryarchaeota (75.6%), Nanoarchaeota (13.9%), Crenarchaeota (6%), Halobacterota (1.7%), Aenigmarchaeota (1%), and Thermoplasmatota (< 1%). The main components at family level for Crenarchaeota and Euryarchaeota were Thermoprotei and Thermococci, respectively.

No assignments at genus level were detected for sites VI and VCF. The archaeal community of VC sediment was represented only by *AR15* members, while the VPV sediments reported the presence of *Thermococcus* with 90% relative abundance, followed by *Palaeococcus*, *Archaeoglobus*, and *Thermodiscus* (<1%).

### Scanning electron microscopy analysis

**Figure 7:**
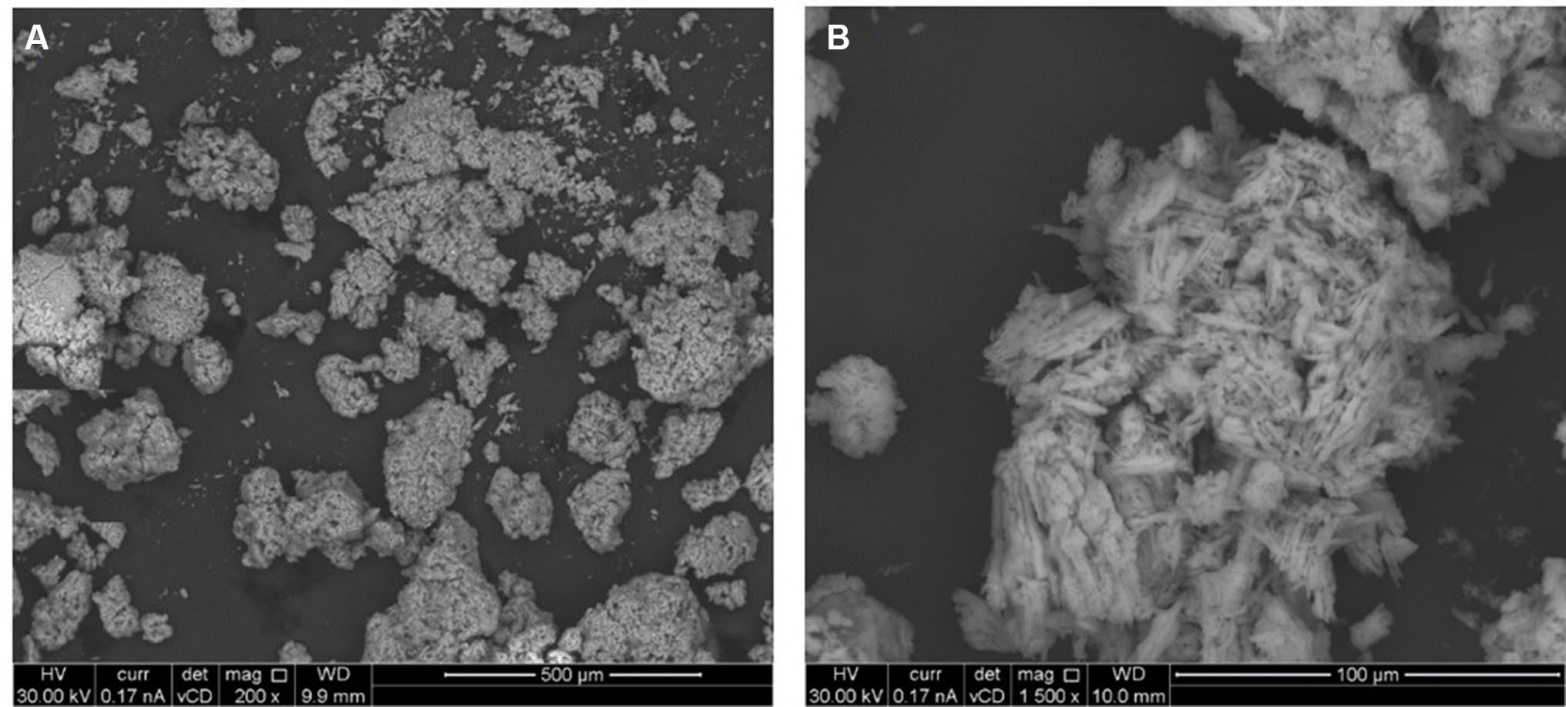
Scanning electron image of floccule surface, performed at two maginification respectively, 200X **(A)** and 1,500X **(B)**.

Figure 7 shows the floccule surface by scanning electron microscopy. The microanalysis (Supplementary Figure S1) showed the predominance, expressed as normalized mass (%), of oxygen in four different oxides (SO_3_, Na_2_O, MgO and SiO_2_), sulfur, carbon, sodium, chlorine, magnesium and silicon content in the sample. The higher amount retrieved in floccule samples was relative to the oxygen content, with a normalized mass percentage of 52.04%, and to the sulfur content with a normalized mass percentage of 32.34%. All other elements were present in percentages higher than 0.2% of normalized mass, as listed in Table 2.

**Table 2:** Main chemical elements detected in floccule sample.

Element	At.No	Mass [%]	Mass Norm. [%]	Comp.	Sto. [%]	Sto. Norm. [%]	Abs. Error [%]	Rel. Error [%]
		110.19	100.00		110.19	100.00		
Oxygen*	8	52.04	47.23		0.00	0.00	19.03	36.57
Sulfur	16	32.34	29.35	SO_3_	80.74	73.27	1.24	3.83
Carbon*	6	8.95	8.12		8.95	8.12	3.82	42.67
Sodium	11	8.73	7.92	Na_2_O	11.77	10.68	0.69	7.92
Chlorine	17	7.38	6.70		7.38	6.70	0.31	4.25
Magnesium	12	0.55	0.50	MgO	0.91	0.82	0.08	14.92
Silicon	14	0.21	0.19	SiO_2_	0.45	0.41	0.05	21.67

At. No., atomic number; mass %, mass percentage; normalized mass%, normalized mass percentage; Comp., composition; Sto., stechiometric ratio; Sto. Norm., normalized stechiometric ratio; abs. Error (%), absolute error; rel. Error (%), relative error.

* relative error > 25%.

### Statistical analysis

PERMANOVA analysis revealed significant differences between microbial assemblages from sediments, both at the phylum and genus levels (Supplementary Table S2). In particular, the microbial communities were significantly different at the phylum level between sediments of VC and VI, while at the genus level between sediments of VC *vs* VI and VPV, as well VI *vs* VPV. As shown by the SIMPER test, dissimilarities between bacterial communities were mainly due to Proteobacteria (13.93%) and Bacteroidota (11.08%) at the phylum level. *Campylobacter* and *Sulfurovum* genera contributed to the dissimilarities between bacterial assemblages of VPV vs. VC and VI. Not assigned taxonomic groups at the genus level were also responsible for dissimilarities between sediment microbial communities of VC and VPV, as well as VC and VI (Supplementary Table S3).

**Figure 8:**
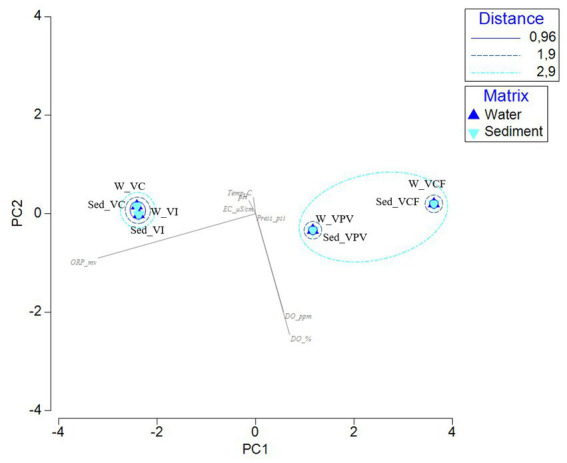
Principal Component Analysis computed on the physico-chemical parameters measured in the sample sites. [ORP_mv, Oxygen Redox Potential (mv); Temp_C, temperature (°C); pH, pH; EC_μS/cm, Electrical conductivity (μS/cm); Press_psi, pressure; DO_ppm, Dissolved Oxygen (ppm); DO_%, Dissolved Oxygen (%)].

The PCA computed on the dataset of physicochemical data highlights the spatial separation of samples. The two main components explained 99.3% of the total variance, with the first component mainly represented by the ORP values (negative correlation), while the second component mainly expressed by the Dissolved Organic (DO) (negative correlation). In Figure 8 water and sediment samples from VC and VI sites are grouped together with a minimum distance, while water and sediment samples from VCF and VPV formed two separate sub-cluster included in a bigger group.

**Figure 9:**
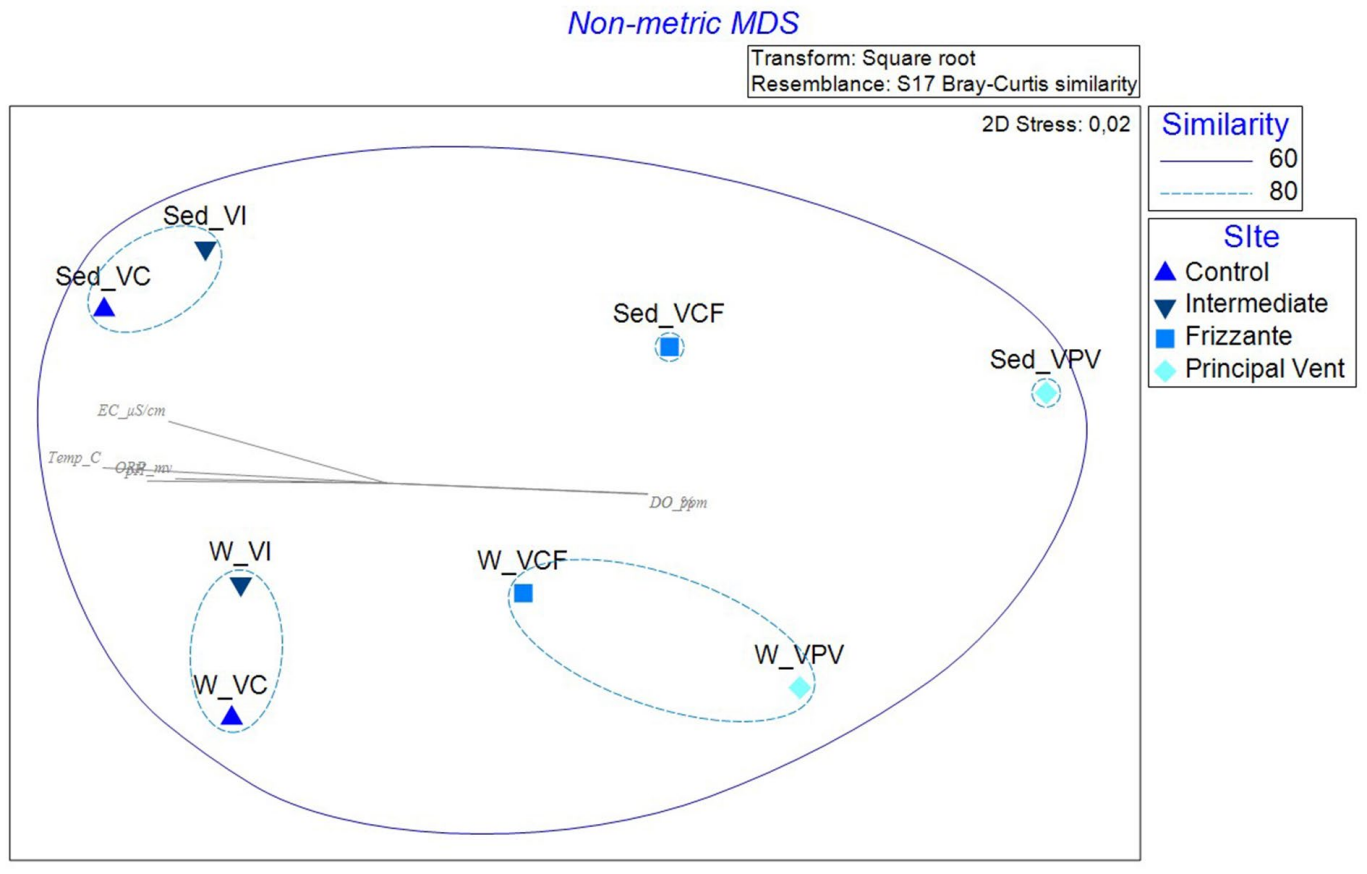
Non-metric multidimensional scaling analysis (nMDS) computed on transformed and clustered abundance data for bacterial communities retrieved in water and sediment samples. pH, pH; Temp_C, Temperature (°C); ORP_mv, Oxygen Redox Potential (mv); EC_μS/cm, Electrical conductivity (μS/cm); DO_ppm, Dissolved Oxygen (ppm); DO_%, Dissolved Oxygen (%).

The nMDS computed on relative abundances at the phylum level is shown in Figure 9. The picture shows a different distribution of water and sediment samples (respectively on the top and bottom), and a sort of gradient from VC samples to VPV samples from the left to the right side. All samples showed a similarity of 60%, but three subclusters including samples similar for 80% are formed, as follows: sediment samples from VI and VC sites, water samples from VI and VC sites, water samples from VCF and VPV sites.

The Simper analysis computed by setting sampling site as factor confirmed a high intragroup similarity, ranging from 72.5 to 79.8% (groups Vulcano Control and Vulcano Campo Frizzante, respectively), and highlighted the highest dissimilarity percentages between Vulcano Control and Vulcano Principal Vent groups, accounting for 44.2%. The dissimilarity value is mainly attributed to the relative abundance of Plancomycetota and Nitrospirota, with cumulative contribute percentages of 67.9 and 70.1%.

## Discussion

Hydrothermal environments are considered among the most hostile environments on our planet, inhabited by organisms that have developed physiological strategies to cope with unfavorable environmental conditions. The presence of high temperatures, negative redox potential and low pH values as a result of strong CO_2_ emissions contribute to shaping the entire ecosystem, determining also important shifts in the structure of the microbial communities, which are really sensitive and responsive to environmental changes. The hydrothermal vents are considered natural laboratories for the study of important processes such as ocean acidification and global warming (González-Delgado and Hernández, 2018; Rizzo et al., 2022). However, this aspect has been well addressed mainly in previous studies focused on the effects of “*hydrothermalism”* on living communities of seagrass (Apostolaki et al., 2014; Vizzini et al., 2019), fish and benthic organisms (Mirasole et al., 2017). To date, studies concerning the microbial communities inhabiting acidified environments have increased, but in many cases the knowledge about the influence of the environmental parameters on the taxonomic structure of microbial communities is still fragmented. This is the case of several investigations (Kerfahi et al., 2014; Taylor et al., 2014; Raulf et al., 2015), in which the microbiological analyses were not associated with the measurements of physico-chemical parameters, thus making hard to establish how external conditions might affect the response of microbial communities. The effects of vent fluids on the structure of microbial communities have been investigated in sediments samples from the shallow hydrothermal vents of Panarea Island and in the Pacific Ocean (Manini et al., 2008). The microbial communities of Vulcano Island have been also deeply studied in the past (Gugliandolo and Maugeri, 1993, 1998; Maugeri et al., 2002; Gugliandolo et al., 2003), but has received less attention more recently and in particular within the context of ocean acidification. In this study, the area was characterized by a decrease in pH (up to 2 units) from the VC to the VPV site in line with the redox potential decline. The conditions of pH and redox potential here reported are overall comparable to previous studies (Gugliandolo and Maugeri, 1993, 1998; Maugeri et al., 2002; Gugliandolo et al., 2003) but also to more recent data reported for the same area by Sciutteri et al. (2022).

**Figure 10:**
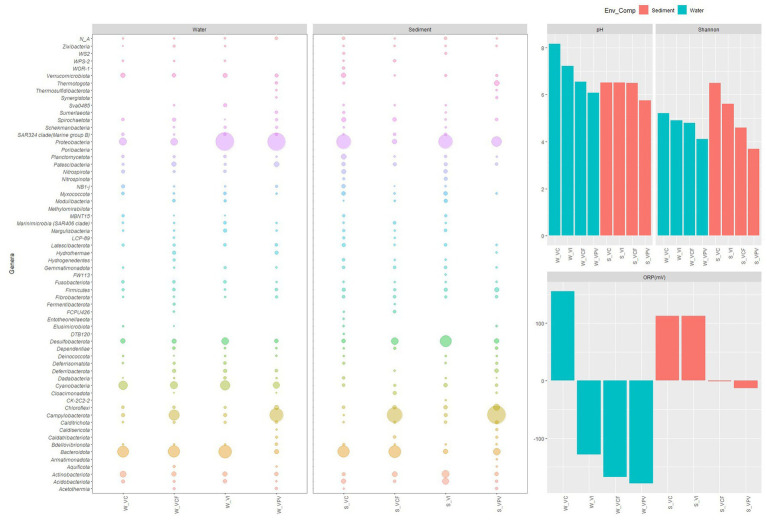
Bubble plot showing relative abundance of phyla in water and sediment samples, related to the pattern of pH, oxygen redox potential and Shannon index.

As shown in Figure 10, the shifts in microbial populations was reflected in lower diversity and richness in more acidified sites, suggesting a shaping of the communities in favor of more specialized taxa, which are able to cope with extreme conditions.

### Bacteria

At phylum level, the taxonomic structure of microbial communities changed in function of the matrix (water or sediment) and of the sampling site, with a general pattern well represented in Figure 10. Overall, water communities were characterized by a predominance of Proteobacteria and Bacteroidota, followed by Cyanobacteria with the only exception of samples from VPV site, where Campylobacterota predominated over Bacteroidota and became the second most abundant group after Proteobacteria. Differently, sediment communities showed more marked shifts moving toward the most acidified sites, as Campylobacterota became predominant over the total bacterial community in VCF and VPV sites. In the water samples, the group of Bacteroidota and Proteobacteria gradually shifted from a relative abundance of 16.2 and 60.3% in the control to 10.1 and 47.3% in the principal vent, respectively. A similar trend was observed in sediment samples, in which Bacteroidota and Proteobacteria abundance decreased from 21.2 and 34.3% to 6.7 and 14.2%, respectively (Figure 10). These findings suggest that in non- or less- acidified sites the microbial structure included the main microbial groups commonly observed in marine waters, while in the most acidified site the microbial population reflected the typical feature of shallow hydrothermal vents, in which the co-occurrence of chemoautotrophs and photoautotrophs/heterotrophs is well documented (Tarasov et al., 2005).

The presence of Proteobacteria and Bacteroidota taxa in sediment samples is in line with previous studies on microbial diversity in hydrothermal sediments of the Tyrrhenian Sea (Maugeri et al., 2010; Gugliandolo et al., 2015; Bortoluzzi et al., 2017). More recently, the structure of microbial communities inhabiting Vulcano Island vents has been investigated by Fagorzi et al. (2019), who denoted the predominance of Actinobacteria, Proteobacteria, and Firmicutes members in soils of different sites characterized by distinctive gas emissions and temperature conditions. In our study, Firmicutes and Actinobacteria were also detected among the main components of the sediment bacterial communities, with higher abundance than in water, but they were not predominant. The group of Campylobacterota showed a strong increase in abundance from the control site to the principal vent site, with a values varying from 0.1 to 26.6% and 57.6 in water and sediment samples respectively, therefore more markedly in sediments. This taxonomic group, formerly classified as the Class Epsilonproteobacteria, includes predominant bacterial primary producers in hydrothermal mixing zones, where these organisms play a significant role in the sulfur, nitrogen and hydrogen fluxes at deep-sea hydrothermal fields (Nakagawa et al., 2005; Campbell et al., 2006; Oren and Garrity, 2021) due to their ability of oxidizing reduced sulfur, formate or hydrogen. The metabolic capacity of this group has been evidenced by its versatility in the use of different metabolic pathways, namely hydrogen-oxidizing sulfur respiration by hydrogenase and polysulfide reductase, sulfur compounds-oxidizing oxygen/nitrate-respiration pathway by the Sox multienzyme system and hydrogen-oxidizing/nitrate reduction pathways, in dynamic and transient environmental conditions (Yamamoto and Takai, 2011).

The presence of Campylobacterota has been documented in sediments from vents in Panarea (Aeolian Arc), Kolumbo (Aegean Sea), Milos (Aegean Sea) (Sievert et al., 1999; Giovannelli et al., 2013; Kilias et al., 2013; Lentini et al., 2014), but there is no evidence of its presence in Vulcano vents from the characterization carried out by Fagorzi et al. (2019). However, in line with our findings, the presence of this taxa have been highlighted recently in a colonization experiment on microbial biofilms in the same area by Sciutteri et al. (2022), which reported that the oxidation of reduced sulfur species is one of the main energy-yielding processes within the biofilm community correlated with CO_2_ and H_2_S availability. This finding is intriguing as despite the Vulcano area is classified as a site of shallow hydrothermal vents, the taxonomic structure of naturally inhabiting water and sediment bacterial communities in the most acidified site reflects the typical features of deep hydrothermal vents.

Punctiform variations between water and sediments were evidenced in the bacterial populations, with some correlated to the relative matrix, as for example the higher representation of Acidobacteriota and Actinobacteriota in sediments than in waters, while Cyanobacteria were more abundant in the water column. Also specific taxonomic groups as Acidobacteria and Actinobacteria showed a diminishing trend in their abundance from the control site to the principal vent. Acidobacteriota members are especially prevalent in soil samples (Kielak et al., 2016; Eichorst et al., 2018) and include some bacteria with unique physiological properties, such as iron reduction capacity (Coates et al., 1999; Rogers et al., 2003) and thermophilic lifestyle (Losey et al., 2013). Kielak et al. (2016) reported that many Acidobacteria members release siderophores to scavenge iron from soil minerals by the formation of Fe^3+^ complexes. The presence of the cation Fe^2+^ was demonstrated in 10 Vulcano hydrothermal fluids with concentrations ranging from 0.02 ppm to 309 ppm, and it has been proved also that reactions in which/or Fe(III) serves as the TEA (namely, the oxidation of CH_4_ to CO_2_ coupled to the reduction of Fe(III) in magnetite to Fe^2+^) releases a great amount of energy (Amend et al., 2003). The presence of bacterial taxa involved in the iron cycle is also supported by the detection of other groups, such as *Deferribacter* (Phylum Deferribacterota) and *Deferrisoma* (Phylum Deferrisomatota). On the other hand, the presence of Cyanobacteria members in the water communities supported the co-occurrence of chemoautotrophs and photoautotrophs/heterotrophs in shallow hydrothermal systems, as this groups includes members able to carry out oxygenic photosynthesis and anoxygenic photosynthesis (Li et al., 2018).

The bacterial populations of sediments were also characterized by higher abundances of Desulfobacterota compared to water samples. This is the only taxonomic group which was more represented in the control and intermediate sites, and decreased in the VCF e VPV sites. This further underlines a community structure based on the different redox processes involving sulfur, with a shift from a more sulfate-reducing based metabolism in less acidified sampling sites (represented by the sulfate-reducing Desulfobacterota) to the sulfur-oxidation in the more acidified sampling points (represented by the sulfur-oxidizing Campylobacterota). Interestingly, some taxonomic groups were retrieved exclusively in samples collected at the VPV site, with higher extent in the sediment samples, up to 2.9% of the total bacterial community in the case of Thermotogota and 1.7% for Deferribacterota. The phylum Thermotogota comprises bacteria commonly retrieved in marine vents and terrestrial hot springs (Nesbø et al., 2015; Foght et al., 2017), with a lifestyle characterized by anaerobic fermentation and thermophilia and hyperthermophilia (Pollo et al., 2015), while Deferribacterota members are capable of anaerobic respiration using iron, manganese or nitrate. More markedly, we found a higher abundance of Chloroflexi members in sediments from the principal vents than in water and in other sediment samples. The presence of thermophilic heterotrophs such as *Thermococcaceae* and *Anaerolineae* was reported in the main hydrothermal vent of the Taketomi submarine hot spring (Yaeyama Archipelago, Japan) with the predominance of thermophilic sulfur oxidizers population (Nunoura et al., 2014).

At genus level, sulfur-oxidizing bacteria (SOB) (phylum *Campylobacterota*) were detected in this study, with the predominance of genus *Sulfuruspirillum* and *Sulfurovum* mainly in water and sediments of VCF and VPV sites. Among the sulfur-reducing bacteria (SRB) 28 different genera have been detected, mostly comprised in the Gammaproteobacteria group (mainly *Desulfobulbus* and *Desulfatitalea*), followed by Desulfobacterota (*Desulforhopalus, Desulfuromusa, Desulfatiglans,* and *Desulfacinum*). Among Gammaproteobacteria, the genus *Thermodesulforhabdus* was also detected in the sediments of the principal vent site. The genus includes Gram-negative, thermophilic, acetate-oxidizing, sulfate-reducing bacterium (Beeder et al., 1995).

As depicted above, in acidified conditions the community structure shifts undergo a change in favor of bacterial taxa previously classified as Epsilonproteobacteria. Interestingly, comparative genomic analysis demonstrated that many Epsilonproteobacteria members share virulence gene and determinants with bacterial pathogens, which benefits them in coping with extreme hydrothermalisms. Evolutionary links between important human/animal pathogens and their nonpathogenic, symbiotic, chemolithoautotrophic deep-sea relatives have been retrieved (Nakagawa et al., 2007). This is in line with a recent study investigating Tor Caldara microbial communities through a metaproteogenomic approach, which demonstrated the presence of *Thiomicrospira*-related Gammaproteobacteria, as well as *Sulfurovum*-related Epsilonproteobacteria, and showed that the expressed pathways were sulfide oxidation and carbon fixation (Patwardhan et al., 2021).

The analyzed colloidal suspensions retrieved in the area revealed a bacterial assemblage constituted by Campylobacterota, Bacteroidota and Proteobacteria, with the associations of sulfur oxidizing *Sulfurovum*, the nitrate-reducing chemolithoautotrophs *Nitratifractor* and *Sulfurimonas* characterized by a flexible metabolism of changing electron acceptors/donors and sources of inorganic carbon, and the *Campylobacter*. This suggest the occurrence of bacterial assemblage with possible trophic association, as observed by Stokke et al. (2015) who found a trophic link between sulfur oxidizing *Sulfurovum* and heterotrophic Bacteroidetes in a hydrothermal chimney biofilm. Interestingly, in the floccules has been found the co-existence of groups involved in the sulfur and nitrogen cycle, which were less abundant in water and sediments. The coupling of sulfur oxidation and dissimilatory nitrate reduction is an important source of energy for the inorganic carbon fixation in hydrothermal systems (Shao et al., 2010). These observation are supported by the microanalysis of elemental composition, which showed a considerable amount of sulfur in the samples.

#### Archaea

The highest and most diversified community of Archaea has been found in the VPV sediments, with a clear abundance of Euryarchaeota, already retrieved in a previous report by Antranikian et al., (2017).

While Crenarchaea and Euryarchea groups were reported to cover the totality of the archaeal community for the area of Vulcano (Gugliandolo and Maugeri, 2019), in our study we identified the phylum of Nanoarcheaota, which appears to be predominant in VI water and sediment, VCF sediment and VC water. In the VPV sediment the groups accounted only for the 13% of the total archaeal community, while Euryarchaeota phylum represented the 75% of the total archaeal community. This could be justified by the more extreme conditions of the sediment, as Euryarchaeota are thermophilic and hyperthermophilic, with strong capacities to cope with extreme high temperatures. The peculiarity of samples could be also the reason for which some archaeal marine groups were not detected in this study, i.e., Thaumarchaoeta and the Marine Group II, despite commonly detected in marine environments as pointed out by Santoro et al. (2019). However, our results are in line with results reported for the same area by Gugliandolo and Maugeri (2019), who found Thermoprotei and Thermococci as main components for Crenarchaeota and Euryarchaeota, respectively.

The metabolic features of Nanoarchaeota members are still not fully known, but they are reported as obligate symbionts of other microorganisms, present small dimension (Huber et al., 2002), and a very small DNA (Waters et al., 2003). Interestingly, a member of the phylum, namely *Nanoarchaeum equitans* from the deep-sea vents of North Iceland (Huber et al., 2002), has been reported for its symbiosis relationship with the crenarchaeon *Ignicoccus hospitalis*, an autotrophic microorganism able to use elemental sulfur as an electron donor and H_2_ as an electron acceptor. The information on the Archaeal community in Vulcano Island is still scant, probably also due to the fact that as they represent a small fraction of the microbial community, and also past studies on these microorganisms in the area of Vulcano island are poor (Antranikian et al., 2017). Anyway, the distribution of the various Archaea phyla is in line with the environmental parameters measured observed in the study area, with greater diversity and abundance at the most extreme VPV site.

In this study, the purpose was to provide a picture of the total prokaryotic community in acidified waters and sediments, but further analysis, more focused on the archaeal community, will be performed by using the support of primers useful to detect specifically the archaeal fraction, as suggested by Wasimuddin et al. (2020).

## Conclusion

The Levante Bay in Vulcano Island is an area of crucial ecological relevance. The results here obtained showed that both in the water column and in the sediments the microbial communities respond very readily to the external changes, and are particularly affected by pH and ORP, which act as the main environmental drivers. Specifically, this was more evident for microbial communities inhabiting sediment than those occurring in water, probably due to the more dynamic conditions of the water column. Gammaproteobacteria and Campylobacterota were the overwhelming predominant bacterial taxa in the most acidified sites, thus confirming their pivotal role in the primary production at shallow hydrothermal vents. The acidification processes shape the microbial communities moving toward an ecological structure based mainly on chemoautotrophy, which supplies energy to the rest of the ecosystem. Based on these results, the microbial community arrangement was different from the previous reports for the same area a few years ago. Moreover, new shifts and ecological dynamics are expected, and are under investigation due to an intensification of pH changes observed in the area. In particular, from October 2021 to date, an intensification of gaseous emissions occurred, as documented by weekly bulletins from the Aeolian Monitoring Center (https://cme.ingv.it/statodi-attivita-dei-vulcani-eoliani/vulcano) indicating the state of maximum alert that hinder the tourist activities of the whole island of Vulcano. Although microorganisms prove to be valuable indicators in such dynamic ecosystems, future studies should be focused on the effects of microbial structure variations on the subsequent trophic levels.

## Data availability statement

The datasets presented in this study can be found in online repositories. The names of the repository/repositories and accession number(s) can be found at: https://www.ncbi.nlm.nih.gov/, PRJNA979234.

## Author contributions

CR and EA contributed to the conception and design of the study, and analyzed all data. CR, EA, and RC conducted experiments and lab analysis. CR, EA, PC, and VS participated in the sample collection. RC provided technical support to the experiment. CR, EA, RC, and FF drafted the manuscript. CR, TR, and FA critically reviewed and revised the manuscript. All authors contributed to the article and approved the submitted version.

## Funding

This study was supported by the projects Marine Hazard funded by the Italian Ministry for University and Research PON “R&C” 2007–2013 PON03PE_00203_1 CUP: B62F15000210005 – Development of innovative technologies for identification, monitoring, and mitigation of natural and anthropic contamination processes.

## Conflict of interest

The authors declare that the research was conducted in the absence of any commercial or financial relationships that could be construed as a potential conflict of interest.

## Publisher’s note

All claims expressed in this article are solely those of the authors and do not necessarily represent those of their affiliated organizations, or those of the publisher, the editors and the reviewers. Any product that may be evaluated in this article, or claim that may be made by its manufacturer, is not guaranteed or endorsed by the publisher.
