# Supplementary material for: Microbial communities inhabiting shallow hydrothermal vents as sentinels of acidification processes
Source: Front Microbiol. 2023 Sep 1;14:1233893. doi: 10.3389/fmicb.2023.1233893 (PMC10505797; doi:10.3389/fmicb.2023.1233893)
Supplement: Supplementary file 1 [file Data_Sheet_1.docx]

Supplementary Material

Microbial communities inhabiting shallow hydrothermal fields as sentinels of acidification processes

Erika Arcadi^1^, Carmen Rizzo^2,3*^, Rosario Calogero^1^, Valentina Sciutteri^1^, Francesco Fabiano^2^, Pierpaolo Consoli^1^, Franco Andaloro^1^, Teresa Romeo^1,4^

^1^ Department of Integrative Marine Ecology, Stazione Zoologica “Anton Dohrn”, Sicily Marine Centre, Contrada Porticatello, 29, 98167 Messina, Italy

^2^ Stazione Zoologica Anton Dohrn–Marine Biotechnology Department, Sicily Marine Centre, Villa Pace, Contrada Porticatello 29, 98167 Messina, Italy; [carmen.rizzo@szn.it](mailto:carmen.rizzo@szn.it)

^3^ Institute of Polar Sciences, National Research Council (CNR-ISP), Spianata S. Raineri 86, 98122 Messina, Italy

^4^ Department of Integrative Marine Ecology, Stazione Zoologica “Anton Dohrn”, Sicily Marine Centre, Via dei Mille 46, 98057 Milazzo, Italy; teresa.romeo@szn.it

^5^ National Institute for Environmental Protection and Research, Via dei Mille 46, 98057 Milazzo, Italy

*** Correspondence:**Corresponding Author
carmen.rizzo@szn.it


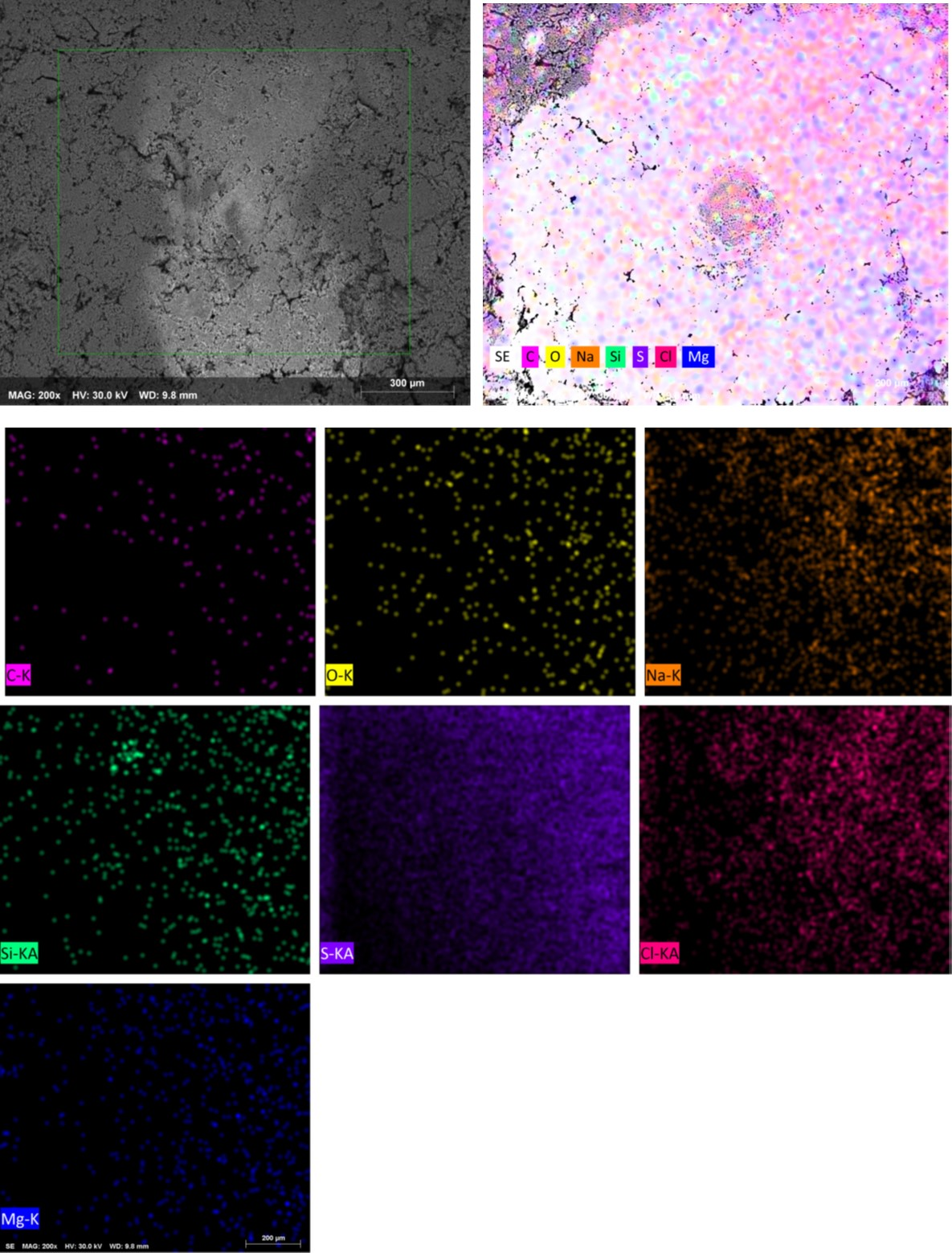


**
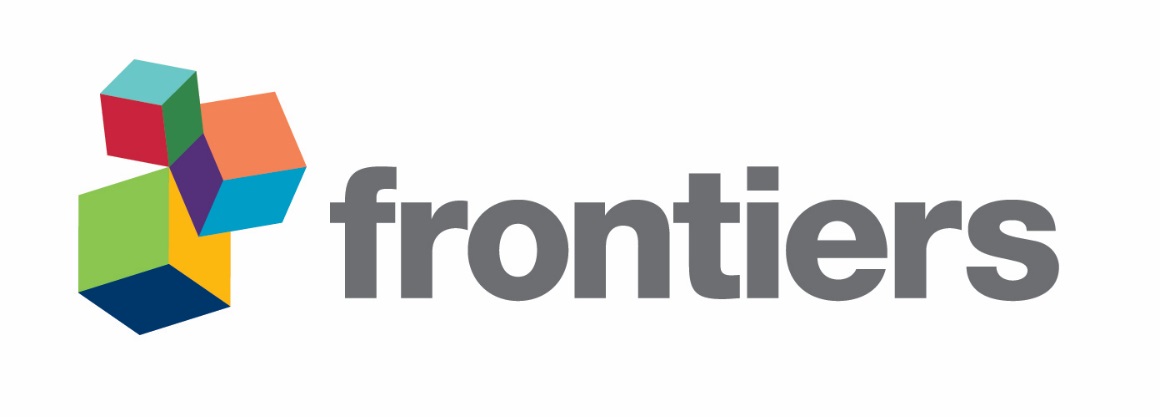
**

**Supplementary Figure 1.** Scanning electron pictures showing the microanalysis mapping of floccule samples. Chemical elements retrieved with percentage higher than 0.2% of the normalized mass are showed with different colors.

**Supplementary Table S1.** Total number of sequence reads, good quality reads, observed numbers of ASVs, Shannon diversity, Simpson and Chao 1 index per sample of the 16S rRNA gene data sets.

|  | Water samples | | | |  | Sediment samples | | | |
| --- | --- | --- | --- | --- | --- | --- | --- | --- | --- |
|  | W_VC | W_VI | W_VFCF | W_VPV |  | Sed_VC | Sed_VI | Sed_VCF | Sed_VPV |
| Total Reads | 104156.3 | 155039.7 | 94081.33 | 22357.3 |  | 149589.7 | 11222.33 | 45340.67 | 89885 |
| Good Quality Reads (%) | 86.7 | 83.5 | 83.2 | 85.5 |  | 83.9 | 78.51333 | 84.07333 | 84.57 |
| ASVs | 71271 | 81574 | 60205 | 132112 |  | 103538 | 5378 | 32530 | 66324 |
| Chao1 | 958.4 | 1426.1 | 1628.8 | 1391.2 |  | 2828.8 | 611.5 | 758.6 | 496.7 |
| Shannon | 5.2 | 4.9 | 4.8 | 4.1 |  | 6.5 | 5.6 | 4.6 | 3.7 |
| Simpson | 0.98 | 0.97 | 0.96 | 0.93 |  | 0.99 | 0.99 | 0.95 | 0.90 |
| Inv Simpson | 86.5 | 78.8 | 34.9 | 15.2 |  | 230.6 | 169.7 | 25.6 | 10.4 |

Table S2. Permanova analysis at the multivariate level on the community assemblages of water (W) and sediment (Sed) samples. Significant results are highlighted. Pair-wise tests were conducted after significant P(perm) results. ”*” indicates statistical significance level (* = significant; *** = highly significant).

| **PERMANOVA** | | | | | | | | | |
| --- | --- | --- | --- | --- | --- | --- | --- | --- | --- |
|  |  | **Source of variation** | **df** | **SS** | **Pseudo-F** | **P(perm)** | **perms** | **P(MC)** |  |
| Archaea | W_Phylum | Site | 3 | 4471.50 | 10.86 | 0.39 | 719.00 | 0.42 |  |
|  | Sed_Phylum |  | 3 | 9242.10 | 19.45 | 0.10 | 685 | 0.14 |  |
|  |  |  |  |  |  |  |  |  |  |
| Bacteria | W_Phylum | Site | 3 | 2544.70 | 0.89 | 0.57 | 954 | 0.53 |  |
|  | W_Genus |  | 3 | 6475 | 10.14 | 0.43 | 966 | 0.46 |  |
|  | Sed_Phylum |  | 3 | 9375 | 29.58 | **0.024** | 955 | **0.029** | * |
|  | Sed_Genus |  | 3 | 15486 | 33.57 | **0.001** | 962 | **0.003** | *** |
|  |  |  |  |  |  |  |  |  |  |
| **PAIR-WISE TEST** | | | | | | | | | |
|  |  | **Groups** |  |  |  |  | **perms** | **P(MC)** |  |
| Bacteria | Sed_Phylum | Sed_VC Sed_VI |  |  |  |  | 10 | **0.016** | * |
|  |  |  |  |  |  |  |  |  |  |
|  | Sed_Genus | Sed_VC Sed_VI |  |  |  |  | 10 | **0.025** | * |
|  |  | Sed_VC Sed_VPV |  |  |  |  | 10 | **0.033** | * |
|  |  | Sed_VI Sed_VPV |  |  |  |  | 10 | **0.037** | * |

Table S3. SIMPER analysis on significantly different bacterial communities at the phylum and genus level sediment (Sed) samples.

| **Phylum** | | | | |
| --- | --- | --- | --- | --- |
| **Average dissimilarity = 62.51** | **Group Sed_VC** | **Group Sed_VI** |  |  |
| Species | Av. Abundance | Av. Abundance | Contrib% | Cum.% |
| Proteobacteria | 170.41 | 39.5 | 13.93 | 13.93 |
| Bacteroidota | 141.55 | 31.49 | 11.08 | 25.01 |
| Actinobacteriota | 95.75 | 18.8 | 8.02 | 33.03 |
| Desulfobacterota | 102.39 | 30.75 | 6.83 | 39.85 |
| Planctomycetota | 53.25 | 6.34 | 4.9 | 44.75 |
| Verrucomicrobiota | 48.55 | 5 | 4.49 | 49.24 |
| Spirochaetota | 41.98 | 2.47 | 3.65 | 52.89 |
| NB1-j | 32.22 | 3.44 | 3.35 | 56.24 |
| Patescibacteria | 38.81 | 6.91 | 3.31 | 59.55 |
| Myxococcota | 37.47 | 8.26 | 3.11 | 62.66 |
|  |  |  |  |  |
| **Genus** | | | | |
| **Average dissimilarity = 76.66** | **Group Sed_VC** | **Group Sed_VI** |  |  |
| Species | Av. Abundance | Av. Abundance | Contrib% | Cum.% |
| NA | 195.45 | 49.67 | 5.92 | 5.92 |
| Woeseia | 90.60 | 17.17 | 3.11 | 9.03 |
|  |  |  |  |  |
| **Average dissimilarity = 81.34** | **Group Sed_VC** | **Group Sed_VPV** |  |  |
| Species | Av. Abundance | Av. Abundance | Contrib% | Cum.% |
| NA | 195.45 | 96.81 | 3.91 | 3.91 |
| *Campylobacter* | 0 | 117.16 | 3.67 | 7.58 |
| *Sulfurovum* | 2.33 | 96.74 | 3.07 | 10.65 |
|  |  |  |  |  |
| **Average dissimilarity = 77.50** | **Group Sed_VI** | **Group Sed_VPV** |  |  |
| Species | Av. Abundance | Av. Abundance | Contrib% | Cum.% |
| *Campylobacter* | 0.75 | 117.16 | 7.94 | 7.94 |
| *Sulfurovum* | 5.96 | 96.74 | 6.6 | 14.54 |
| *Sulfurimonas* | 2.71 | 49.27 | 3.23 | 17.78 |
| NA* | 49.67 | 96.81 | 3.22 | 20.99 |

*NA, not assigned.
